# Analysis of technology acceptance and planned behavior of using shared E-biked to support the sustainable transportation development goal in smart cities

**DOI:** 10.3389/fpsyg.2025.1551522

**Published:** 2025-09-04

**Authors:** Sheng-Ming Wang, Zi-Han Xu

**Affiliations:** ^1^Department of Interaction Design, National Taipei University of Technology, Taipei, Taiwan; ^2^Doctoral Program in Design, College of Design, National Taipei University of Technology, Taipei, Taiwan

**Keywords:** shared e-bikes, net zero emissions, structural equation model, theory of planned behavior, smart city

## Abstract

This study offers a thorough investigation into shared electric bicycles (E-bikes) and their role as a key element in advancing sustainable transportation within smart cities. Previous studies have predominantly employed the Technology Acceptance Model (TAM) or the Theory of Planned Behavior (TPB) to investigate user acceptance, yet few have integrated both frameworks to explore how environmental objectives—such as net-zero emissions (NZE)—influence behavioral intention in smart cities. To address this limitation, the present study incorporates key variables including subjective norms (SN), perceived usefulness (PU), user attitudes (ATU), and perceived behavioral control (PBC), while introducing NZE as a factor in user behavior analysis. Data were collected from 298 urban residents aged 18–54 via an online questionnaire. Structural equation modeling analysis was conducted to examine the relationships among SN, PU, ATU, PBC, and NZE. The results showed that SN significantly predicted PU, which confirm the strong influence of social factors on user perceptions. PU stands as a mediating variable, and it significantly influenced users’ ATU and NZE, which reflected that users highly value the convenience and environmental benefits of shared e-bikes. In addition, PU is a key factor in users’ understanding and adoption of NZE, demonstrating that a well-designed shared e-bike system can enhance environmental sensitivity and awareness of green behavior. Furthermore, NZE exerted a significant positive impact on PBC, indicating that greater environmental awareness enhances users’ sense of behavioral control, thereby actively motivating them to opt for shared e-bikes. These findings provides empirical evidence and strategic directions for policymakers and urban planners: by strengthening social advocacy, optimizing system usefulness design, and guiding environmental awareness through green policies, cities can more effectively encourage the adoption of shared electric bicycles in smart urban environments.

## 1 Introduction

Transportation contributes most to city air quality (Yang et al., 2015), which influences animals in the region, climate, and city environment (Makido et al., 2012). Since sharing economy has a positive impact on transportation (Standing et al., 2019), and bicycles can be environmentally friendly, cost-effective, and convenient for short-distance trips, shared bicycles emerged and gained acceptance in cities (Ricci, 2015). The shared bicycle system can replace long-distance walking and short-distance car travel, effectively reducing congestion on urban roads (Samet et al., 2018). Bicycling is an active mode of mobility, an important topic for sustainable city transportation (Cruz and Paulino, 2022). Electric bicycles (E-bikes) enhance the advantages of traditional bikes with a smoother experience and ability to travel longer distances (Kazemzadeh and Ronchi, 2022). Studies have shown that commuting with electric bicycles is pleasant and useful to many people (Jones et al., 2016). Electric bicycles offer a freer way of traveling to people with individual capability and mobility limitation (MacArthur et al., 2017). It has even convinced families to be car-free families. So, shared electric bicycles have emerged (Galatoulas et al., 2020). Shared E-Bikes are slowly spreading and becoming a routine in most countries of the world (Campbell et al., 2016). City transformation into smart cities has a major correlation with shared electric bicycle service mode and added technology and features (Zhang et al., 2021a). Research findings show that if the shared electric bicycle service mode has been introduced, it can reduce CO2 emissions, reduce noise in the city, and be an auxiliary means for city transportation (Pierce et al., 2013). Shared e-bikes are a core component of global decarbonization of transport, reducing carbon emissions and realizing the NZE target (Küfeoğlu, 2024). As a connected transport mode in a smart city, e-bikes generate vast traffic and user information. These pieces of information have the potential to enable city managers to plan transport infrastructure better, dynamically assign resources based on demand, and continuously enhance the quality of urban mobility services (Junaid and Ferretti, 2024). Research further indicates that electric bicycles can cut down substantially the amount of polluted air inhaled by cyclists while cycling (Sweeney et al., 2018). While electric bicycles have numerous advantages, studies have shown that cyclists still have a strong preference towards conventional bicycles compared to electric bicycles in external transport (MacArthur et al., 2017). In contrast, other research demonstrates that there is a strong correlation between frequency of cycling, favorable attitudes towards cycling, and availability of bicycles in households (Zhang et al., 2016). Nonetheless, despite the increasing availability of in-place schemes for E-bike sharing, minimal research has utilized an integrated combination of behavioral and technology acceptance models in examining user adoption in the special setting of smart cities (Li W, et al., 2023). While TAM and TPB have, respectively, prevailed in existing work in shaping user behavior, their integration to examine user behavior regarding sharing usage of electric bicycles in the context of smart cities is rather less common in current studies (Pan et al., 2022). Furthermore, few studies have addressed how total sustainability goals like net-zero emissions (NZE) affect individual-level behavior choices (Wilson and Whitmarsh, 2025). With the objective of filling these research gaps, this research presents an integrated TAM–TPB model with NZE as an environmental value factor aiming to capture holistically the behavioral, technological, and policy aspects of adoption of E-bike in smart-city settings.

Therefore, this study will discuss factors influencing users’ attitudes and perceptions towards shared electric bicycles through five dimensions: (1) SN, (2) PU, (3) ATU, (4) NZE, and (5) PBC. The rest of the paper is organized as follows: Section 2 presents the hypothesis development and theoretical framework, including the research hypotheses and conceptual model. Section 3 describes the research methods, covering the data collection procedures, questionnaire design, and analysis factors. Section 4 reports the research results, including descriptive analysis, measurement model, and structural model including path analysis. Section 5 discusses the research results. Finally, Section 6 summarizes the paper and proposes directions for future research.

## 2 Hypothesis development and theoretical framework

There have been a number of pro-environmental behavior studies based on the Theory of Reasoned Action (TRA; Ajzen and Fishbein, 1975) to analyze the behavioral intention of individuals, for instance, the effect of price volatility on consumers’ intention to buy environmentally friendly products (Rehman et al., 2024). Some scholars have suggested that there are shortcomings in the theory and it cannot offer full explanation of people’s volitional behaviors (Kippax and Crawford, 1993). TPB was developed by American researchers Fishbein and Ajzen on the basis of TRA. It effectively addresses shortcomings and is predominantly used to predict and study human behavior (Ajzen, 1991).

Research has established that TPB components directly influence behavioral intentions (Tee et al., 2023), testifying to their significant contribution in this area. Furthermore, the study (Duong et al., 2024) supports that TPB components, i.e., ATU, SN, and PBC, are determinants of behavior. Subjective norms tend to direct individual behavior, employing social relations to shape personal attitudes, moral norms, obligations, and duties to induce healthy behaviors (Arkorful, 2022). Attitude consists of “behavioral beliefs” and “outcome evaluations” and is combined with subjective norms to affect behavior intentions and actions (Li et al., 2020).

ATU, SN, and PBC were also utilized in the interpretation of the intention of the user to use shared bicycles within the same research study (Shakya et al., 2024), with a positive influence. The variables verify the application of TPB in shared E-bike research (Li et al., 2022).

TAM is a central theory for explaining and predicting the adoption of new technology and innovative solutions by users (Davis et al., 1989). The theory finds extensive applications in diverse research areas In a research on the adoption of e-government services among Indian urban citizens, researchers developed a Technology Acceptance Model that integrated the determinant of “preference,” thereby extending the application of the theory (Samuel et al., 2020) TAM is also widely applied in researching and examining attitudes toward modes of transportation (Li X, et al., 2023). The model has also been utilized in previous studies to predict the determinants of user attitudes (Teo et al., 2008). The current research employs ATU and PU, where PU and perceived ease of use (PEOU) together impact ATU.

### 2.1 Research hypotheses

This study integrates TPB and TAM to examine how subjective norms (SN) influence perceived usefulness (PU; SN → PU), how PU influences user attitudes (ATU; PU → ATU), and how PU is also related to individuals’ awareness of net-zero emissions (NZE; PU → NZE), which further reinforces their perceived behavioral control (PBC) in utilizing shared E-bikes (NZE → PBC). Through these relationships firmly established, the model fills the gap between users’ technology perceptions and social factors and overall sustainability targets, providing a concrete analytical framework for the explanation of low-carbon transportation behavior.

SN highlights the role of the social environment in personal travel choices of sustainability (Shi and Jiang, 2023). SN are “the individual’s perception of the importance of others’ wishes or expectations for him or her to behave in a certain way” (Moan and Rise, 2006) and predict PU strongly. Previous studies prove that perceived social pressure has a strong influence on individual behavior (Choi and Chung, 2013). In the same scenarios, the same attributes exist: friend and family attitudes about shared E-bike use, social support for environmental and low-carbon travel, and peer prevalent adoption of low-carbon modes of travel are all significant points for personal travel choice. This concept is validated by research outcomes (Song et al., 2022b).

Accordingly, the first hypothesis (H1) is proposed as follows:

*H*1: Subjective norms(SN) positively influence perceived usefulness(PU).

TAM is an overarching theoretical model for explaining and predicting users’ acceptance of new technologies and innovative products (Davis et al., 1989). It has been used extensively in studies examining transportation mode perception (Li X, et al., 2023), and has been used widely for predicting determinants of user attitude (Teo et al., 2008). ATU and PU are central to the current study, with PU and perceived ease of use (PEOU) both influencing ATU. There are instances in the literature, however, that have established PU is more influential on behavioral intentions than PEOU (Ariff et al., 2012). In the literature, it is established that people’s PU and PBC of shared E-bike services have a positive influence on people’s actual usage behavior (Song et al., 2022a). Attitudinally, there are some studies that have examined people’s identification with and perception of shared E-bike services (Bardi et al., 2019), and it is established that positive attitude is associated with the perception the travel mode provides a pleasant experience and accommodates short-distance travel needs (Su et al., 2024).

The current research investigates users’ responses to the PU of e-bike products, where PU shapes their ATU toward adopting shared e-bikes.

Thus, this study proposed the second and third hypothesis as follows:

*H*2: Perceived usefulness(PU) positively influences user attitudes(ATU).

The research integrates environmental issues into bicyle-sharing studies, considering how the acceptance and use rates of shared bicycle users can be enhanced (Zhu et al., 2020). Existing literature confirms that perceived usefulness exerts a great influence on individuals’ pro-environmental intentions and behaviors (Rezvani et al., 2015). Moreover, shared electric bicycles are widely accepted as a convenient low-carbon way of transportation to accelerate urban decarbonization (Cherry et al., 2009). Therefore, the more useful users perceive shared E-bikes to be, the more they are likely to believe in the role played by such services in achieving net-zero emissions. NZE is a condition where the amount of greenhouse gases emitted into the atmosphere is equal to the amount removed from the atmosphere (Wang and Priyadarshi, 2024). Empirical studies of this nature also study how users’ level of environmental concern relates to their respective behavior. It illustrates that environmental concern is a strong predictor of such behavior, including “Lifestyle Transition” elements in the context of net zero emission (Daziano and Bolduc, 2013). Additionally, related studies have stated that individuals’ knowledge about global warming and green issues, and identification of low-carbon modes of travel, have a positive effect on the choice of green modes of transportation (Ogunbode et al., 2019). In the assumed model, NZE acts as an important mediator between PU and PBC. That is, the model considers how users’ perceptions of the usefulness of shared electric bicycles enable their perception of NZE. Such greater environmental value, in turn, enhances users’ perceived behavioral control over shared E-bike use, making their decision process in favor of low-carbon travel behavior easier.

Notably, personal eco-awareness impacts the preference for electric bicycle travel, as a strong sense of environmental responsibility encourages the adoption of zero-emission modes (Bai et al., 2020). Thus, this study proposed the fourth hypothesis as follows:

*H*3: Perceived usefulness(PU) positively impacts the concept of net zero emissions(NZE).

*H*4: Net zero emission(NZE) positively influences perceived behavioral control(PBC).

### 2.2 The conceptual model

**Figure 1:**
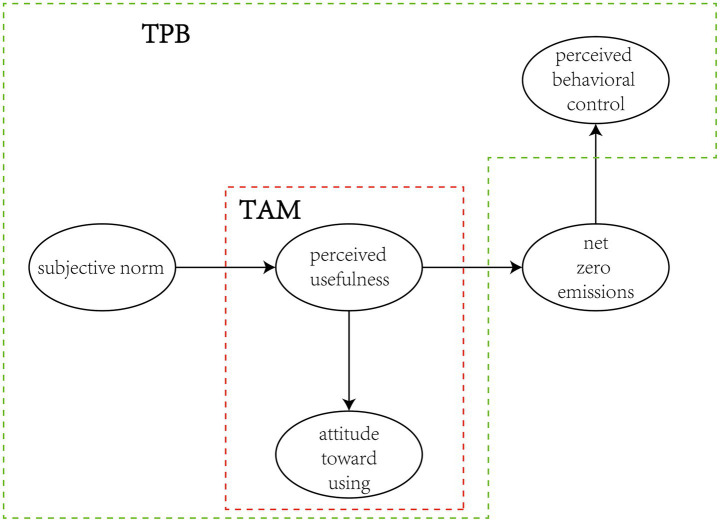
Conceptual model for TPB and TAM integration.

Based on the above review and hypotheses, this research applies Structural Equation Modeling (SEM) to examine the determinants of shared electric bicycle users’ ATU and PBC. To this purpose, the integration of TAM and TPB is employed to develop an explanatory model of user behavior. As TAM concentrates on users’ cognitive beliefs regarding technology emphasizing PU and ATU, TPB emphasizes social and psychological factors such as SN and PBC. With the application of the two frameworks’ combination, the model examines technological acceptance and social influence determinants concurrently, which are crucial to consider to explain behavioral intentions in shared electric bicycle use in smart cities. The integrative model captures the complex interplay between individual judgments of technological effectiveness and the immediate social setting, thus enhancing explanatory power for low-carbon mobility behavior. Figure 1 illustrates the conceptual framework of the study:

The research is on sustainable transportation means in smart city urban planning focusing on shared e-bike systems. The research will cover the following perspective:

1. Attitude (ATU): This involves the comments on individual acceptance and expectation of standard e-bike services and attitudes towards their usefulness for short trips and delivering an acceptable experience.
2. Subjective Norm (SN): This part discusses the influence of friends, family, and societal norms on using shared e-bike services, emphasizing the role of environmental advocacy and low-carbon travel practices.
3. Net Zero Emissions Value (NZE): Addresses the impact of net-zero emissions goals on choosing green transportation options, considering global warming and the importance of reducing carbon emissions.
4. Perceived Usefulness (PU): Focuses on the perceived benefits of shared e-bike services, such as cost-effectiveness compared to other transport modes, fulfilling travel needs, reducing air pollution, and offering diverse travel options.
5. Perceived Behavioral Control (PCB): Individual readiness to choose common e-bike services for short trips, freedom of using the services, adequacy of city traffic conditions for e-bikes, and the individual’s capability (time and energy) to use e-bikes.

The study aims to utilize structural equation modeling to better comprehend the complex relationships of these variables and how they quantify influence toward sustainable travel behavior. The findings of the research will be an addition to sustainable transport research and the achievement of SDG Goal 13.

## 3 Methodology

### 3.1 Data collection

The anonymous online survey consisted of a sample size of 505. The questionnaire was released mainly via the third-party website “Wenjuanxing,” a large and reputed internet surveying site, and specifically targeted respondents in coastal cities, followed by inland cities. In order to ensure the quality of the data, questionnaires containing conflicting responses (including reverse-coded questions) or with inappropriately short completion times were screened out. The resultant dataset consisted of 298 valid questionnaires, with an effective response rate of about 59%.

A 7-point Likert scale was applied to all the questionnaire items except gender and age group, which were asked for separately. To avoid bias or psychological harm, all the questionnaire items were specially phrased to avoid suggestive information or wording that would lead to distress, adhering strictly to the academic ethical norms. The Declaration of Helsinki ethical guidelines (World Medical Association, 2013) were upheld in this study, prioritizing the protection of participants’ rights and welfare.

### 3.2 Analysis factors and questionaire

This study adopts a questionnaire survey method with questions modified from the research questionnaires of the studies of Li X, et al. (2023) to identify factors influencing individuals’ ATU for riding shared E-bikes and their PBC. In this study, the result of the survey questions captured data on five observation variables: subjective norms (SN), perceived usefulness (PU), attitudes (ATU), net zero emission (NZE), and perceived behavioral control (PBC). The socio-demographic information, respondents’ gender, and age were also captured in the data collection. The analysis factors and questionnaire structure for conducting this study are in Table 1.

**Table 1:** Measurement items and questions for each dimension.

Dimension	Item	Question
1. Attitude (ATU)	ATU1	I am looking forward to the shared e-bike service
	ATU2	I think the provision of shared e-bike services can help me complete short-distance purposes
2. Subjective Norm (SN)	SN1	My friends and family’s attitudes towards shared e-bike services will affect my use of the services
	SN2	Most people around me use low carbon dioxide travel
	SN3	The environmental protection and low-carbon dioxide transportation advocated by society make me use shared e-bike services
	SN4	Someone once preached to me about traveling in a low-carbon dioxide and environmentally friendly way
3. Net Zero Emissions Value (NZE)	NZE1	Net zero emissions guide me to choose green travel
	NZE2	Considering global warming, I think energy conservation, emission reduction, and low-carbon dioxide travel are the right choices
	NZE3	I think we should do our part to reduce carbon dioxide emissions
	NZE4	I often pay attention to environmental issues, and I feel worried when I hear about serious environmental problems caused by carbon dioxide emissions
4. Perceived Usefulness (PU)	PU1	I think shared e-bike services are cheaper than other forms of transportation
	PU2	I think shared e-bike services can fully satisfy
	PU3	I think shared e-bike services can reduce air pollution
	PU4	I think the shared e-bike service has enriched my travel options
5. Perceived Behavioral Control (PBC)	PBC1	The use of shared e-bike services is entirely at my discretion
	PBC2	The traffic conditions in my city are suitable for shared e-bike travel
	PBC3	I have enough energy and time to use a shared e-bike to travel

The survey collected information on individuals’ attitudes towards shared electric bicycles and net zero emissions. The final survey had 17 questions, and each of these questions was rated on a 7-point Likert scale, from 1 as ‘Strongly Disagree’ to 7 as ‘Strongly Agree’. An introduction was given to explain shared electric bicycles and net zero emissions so that the respondents could understand the special meanings of the research’s most important observational variables.

## 4 Results

This study employed covariance-based Structural Equation Modeling (CB-SEM) with AMOS 22.0 for rigorous testing and verification of the proposed theoretical model. CB-SEM is best suited to test high methodological rigor relationships between latent constructs because it provides rich model fit indices and measurement error corrections. Given that this study seeks to test various causal effects between the extended TAM, the NZE factor, and TPB constructs, the use of AMOS enables simultaneous estimation of complex interrelationships and testing of the general model fit. In this case, therefore, the use of CB-SEM contributes to the accuracy of the analysis and enhances the reliability and validity of the empirical findings.

### 4.1 Questionnaire survey and data

The study was conducted anonymously online with a total of 505 participants. The final dataset comprised 298 valid responses. Regarding demographics, 24.50% of the respondents were aged 18–24, 40.60% were between 25 and 34, 28.50% were aged 35–44, 6.00% were between 45 and 54, and 0.40% were aged 55 and above. These results indicate that the sample predominantly represents young and middle-aged groups who tend to be more engaged with sustainable mobility topics. In terms of gender, 49.72% of the respondents were male and 50.28% were female, indicating a balanced distribution. Furthermore, the final sample size (*n* = 298) exceeded the commonly recommended minimum standard of 5–10 respondents per project (Hair et al., 2013), meeting the basic requirements for conducting CB-SEM using AMOS (Table 2).

**Table 2:** Statistical analysis of respondent sample.

Age	Frequency	Percentage	Cumulative percent
18–24	73	24.50%	24.50%
25–34	121	40.60%	65.10%
35–44	85	28.50%	93.60%
45–54	18	6.00%	99.60%
Over 55 years old	2	0.40%	100%

### 4.2 Measurement model

The study applied Structural Equation Modeling (SEM) in measuring and testing the structural model through the Maximum Likelihood Estimation (MLE) procedure (Kline, 2023).

All the items possess standardized factor loadings greater than the suggested value of 0.70, which means that every one of the observed variables is capturing its respective construct well (Fornell and Larcker, 1981). Furthermore, Composite Reliability (CR) for all of the constructs is considerably greater than the cutoff value of 0.70, and the Average Variance Extracted (AVE) values for all are greater than the widely accepted cut-off value of 0.50. All these results confirm that the instrument possesses good convergent validity and internal consistency. On the whole, the final format of the questionnaire is in tune with the foregoing theoretical conceptualization and hypotheses, in favor of the adequacy of the measurement model employed to this study (Table 3).

**Table 3:** Result of reliability and validity statistics.

Latent variable	Observed variable	SFL(t)	CR	AVE	SFL range
SN			0.861	0.601	**0.751–0.829**
	sn1	0.751(14.491)			
	sn2	0.759(14.702)			
	sn3	0.786(15.463)			
	sn4	0.829(16.695)			
ATU			0.784	0.585	**0.717–0.812**
	atu1	0.812(14.377)			
	atu2	0.717(12.621)			
PU			0.853	0.579	**0.739–0.794**
	pu1	0.794(15.615)			
	pu2	0.756(14.566)			
	pu3	0.739(14.086)			
	pu4	0.778(15.161)			
NZE			0.859	0.6	**0.754–0.801**
	nze1	0.779(15.233)			
	nze2	0.779(15.242)			
	nze3	0.754(14.552)			
	nze4	0.801(15.853)			
PBC			0.855	0.637	**0.761–0.845**
	pbc1	0.806(15.837)			
	pbc2	0.845(16.954)			
	pbc3	0.761(14.608)			

The square root of the Average Variance Extracted (AVE) for each construct (bold diagonal values) should be greater than its correlations with other constructs (off-diagonal values).

The measurement model possessed adequate descriptive statistical characteristics for all of the observed variables that were associated with their respective latent constructs. In other words, the item means were between 4.25 and 4.51, indicating overall positive participant responses to the measurement constructs. The standard deviations were moderate, and they captured adequate variances in responses that allow valid data analysis. The skewness and kurtosis measures of all of the items fell within the conventional ±1 threshold, indicating nearly normality of the data and satisfying normality assumptions for SEM (Table 4).

**Table 4:** Results for the measurement model.

Latent Variable	Observed variable	M	SD	SK	KU	SE	SMC
SN	sn1	4.43	1.579	−0.213	−0.859	0.368	0.564
	sn2	4.3	1.539	−0.13	−0.925	0.357	0.576
	sn3	4.39	1.577	−0.268	−0.744	0.355	0.618
	sn4	4.36	1.618	−0.188	−0.826	0.36	0.687
ATU	atu1	4.34	1.59	−0.185	−0.822	0.404	0.66
	atu2	4.33	1.572	−0.053	−0.849	0.399	0.515
PU	pu1	4.45	1.489	−0.064	−0.825	0.337	0.63
	pu2	4.38	1.463	−0.14	−0.708	0.339	0.572
	pu3	4.48	1.558	−0.2	−0.731	0.362	0.546
	pu4	4.51	1.566	−0.25	−0.801	0.361	0.605
NZE	nze1	4.47	1.535	−0.098	−0.764	0.35	0.607
	nze2	4.46	1.543	−0.073	−0.828	0.353	0.608
	nze3	4.44	1.569	−0.242	−0.69	0.364	0.568
	nze4	4.38	1.596	−0.172	−0.964	0.359	0.641
PBC	pbc1	4.32	1.635	−0.067	−0.992	0.373	0.649
	pbc2	4.28	1.616	−0.149	−0.9	0.361	0.715
	pbc3	4.25	1.538	−0.051	−0.892	0.356	0.578

Furthermore, the squared multiple correlations (SMC) of the measured variables were also greater than the minimum acceptable value of 0.5, indicating that the indicators appropriately measured the variance of their respective target latent factors and thus possessed strong convergent validity and reliability. The relatively small standard errors (SE) also attest to the precision of mean estimates, lending validity to the measurement findings. Together, the findings guarantee the measurement instrument possesses acceptable internal consistency and construct validity and hence is sufficient for subsequent structural model testing and hypothesis testing (Table 5).

**Table 5:** Fornell-Larcker discriminant validity.

Construct	A	B	C	D	E
A. SN	**0.775**				
B. ATU	0.617	**0.765**			
C. PU	0.652	0.648	**0.761**		
D. NZE	0.689	0.676	0.697	**0.775**	
E. PBC	0.604	0.68	0.69	0.604	**0.798**

The square root of the Average Variance Extracted (AVE) for each construct (bold diagonal values) should be greater than its correlations with other constructs (off-diagonal values).

The discriminant validity was verified against the requirements that the square root of the AVE of each construct should be greater than its correlations with all other constructs, thereby yielding sufficient distinctiveness between the latent constructs. As it can be seen from the table, the diagonal values are the square roots of the AVE of each construct, and all are greater than the inter-construct correlation coefficients. The result indicates that the measurement indicators of each construct possess sufficient discriminant validity, and no conceptual overlap or confusion exists.

### 4.3 Structural model and path analysis

This model was path-analyzed using AMOS 22.0. The goodness-of-fit was assessed overall by multiple fit indices. In particular, the chi-square to degrees of freedom ratio (χ^2^/df) was 1.92, which was between 1 and 3 and reflects an acceptable fit of the model. The goodness-of-fit index (GFI) was 0.919, which was above the cutoff of 0.90. The Standardized Root Mean Square Residual (SRMR) was 0.0732, and Root Mean Square Error of Approximation (RMSEA) was 0.056, both of which were below the cutoff of 0.08, reflecting good fit. Incremental fit indices, like the Normed Fit Index (NFI = 0.92), Tucker-Lewis Index (TLI = 0.952), Comparative Fit Index (CFI = 0.96), Relative Fit Index (RFI = 0.906), and Incremental Fit Index (IFI = 0.96), were all above the cutoff of 0.90, which also reflects adequacy of the model. Furthermore, both Parsimony Normed Fit Index (PNFI = 0.778) and Parsimony Goodness-of-Fit Index (PGFI = 0.69) were above the threshold of 0.50, which reflects acceptable model parsimony. Overall, these results suggest that the structural model has satisfactory overall fit and can be considered robust for further hypothesis testing (Table 6).

**Table 6:** Model fit indices for the structural equation model.

Fit index	Recommended threshold	Estimate	Interpretation
χ^2^ / df	Between 1 and 3	1.92	Acceptable fit
GFI	> 0.90	0.919	Good fit
SRMR	< 0.08	0.0732	Good fit
RMSEA	< 0.08	0.056	Good fit
NFI	> 0.90	0.92	Good fit
TLI	> 0.90	0.952	Good fit
CFI	> 0.90	0.96	Good fit
RFI	> 0.90	0.906	Good fit
IFI	> 0.90	0.96	Good fit
PNFI	> 0.50	0.778	Acceptable
PGFI	> 0.50	0.69	Acceptable

**Figure 2:**
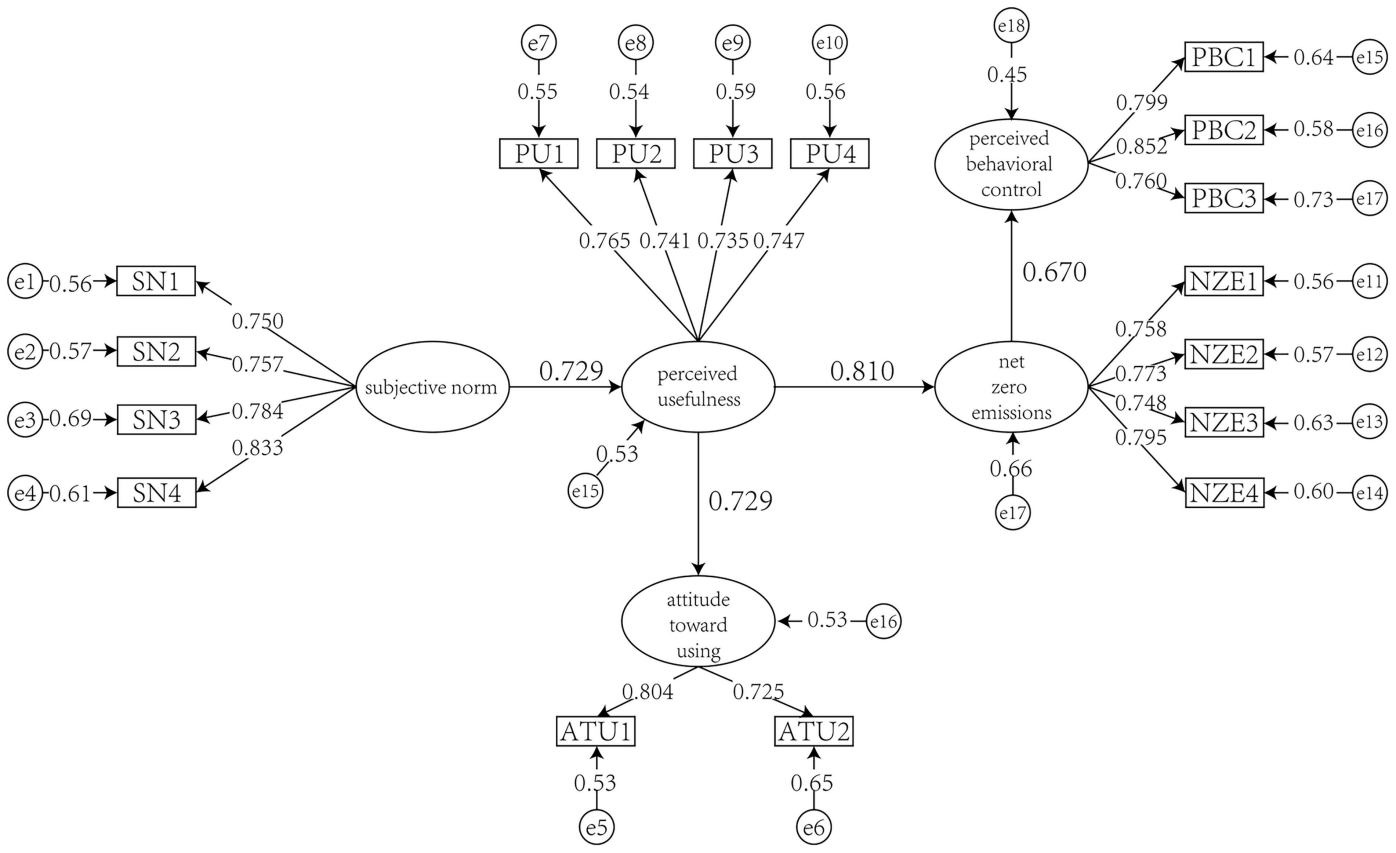
The results of the proposed model.

The structural model results are presented in the Figure 2.

The path analysis results indicated significant positive relationships among the hypothesized constructs. Specifically, Subjective Norms (SN) had a significant effect on Perceived Usefulness (PU; *t* = 9.791, *β* = 0.729), PU significantly influenced Attitude (ATU; *t* = 9.462, *β* = 0.729), and PU also had a strong positive impact on Net Zero Emission (NZE; *t* = 10.594, *β* = 0.810). Furthermore, NZE was positively associated with Perceived Behavioral Control (PBC; *t* = 9.306, *β* = 0.670). All paths were statistically significant at the 0.01 level, confirming the support for H1 through H4 (Table 7).

**Table 7:** Structural model results.

Hypotheses	Path	T-value	β(Path coefficient size)	Results
H1	SN→PU	9.791	0.729	Yes
H2	PU→ATU	9.462	0.729	Yes
H3	PU→NZE	10.594	0.81	Yes
H4	NZE→PBC	9.306	0.67	Yes

## 5 Discussion

Subjective norms would be expected to shape perceived usefulness of shared transport tools among users in line with the current literature (Xu et al., 2023). Users are more likely to perceive shared mobility services in the sharing economy as useful in accordance with peer attitudes and extrinsic social pressures (Zhang et al., 2021b). It is a position that has been supported through studies examining the role derived from individual values and community influence (Bagdatli and Ipek, 2022). It is one of the social environmental factors and plays an important part in shaping the perceived usefulness among users. Users are not just impacted by explicit peer pressure, but also by general sociocultural environments and environmental values, which then shape their perceptions in the context of the usefulness of shared electric bicycles (Steg and Vlek, 2009).

This is an instance where the process indicates that utilization of shared mobility devices is not only based on individual rational thought but also on the interplay between environmental responsibility and social identification, hence a contribution to the TAM model in terms of elucidating the mechanisms of external influence on perceived usefulness. The dominance of subjective norms in the prediction of perceived usefulness indicates that environmental awareness fostered in the community and acceptance by “significant others” can effectively influence users’ perceptions of shared e-bikes’ ease of use (PEOU; Buabeng-Andoh, 2018). Further, electric bicycles (E-bikes) allow for smoother integration with public transport systems, including subways and buses, effectively resolving the “last mile” problem (Horstmannshoff and Redmond, 2024). Such integration makes urban transport more convenient and flexible, ultimately contributing to enhanced travel efficiency. Use of E-bikes also raises the perceived usefulness of users, as it is well known that they view them as convenient mobility aids that meet contemporary transportation requirements. Such convenience is aligned with general lifestyle choices pertaining to sustainable transportation awareness, thereby facilitating social acceptance of shared E-bike services, as SN1, SN2, and SN3 findings suggest. Overall, the use of E-bikes emphasizes their convenience and worth in addressing urban mobility demands.

Perceived usefulness can also have a bearing on people’s attitudes towards the use of shared electric bicycles. This is to say that customers will use shared E-bikes frequently if they perceive that they will be useful for their personal transport. In essence, people make decisions based on whether or not they find a tool or a service useful to them (Teo and Van Schaik, 2012). Regardless of the practices used in adopting attitudes towards the use of E-bikes, both the device and the app must have useful features because perceived usefulness is the key to determining long-term behavior adoption (Pasca et al., 2024).

In particular, the outcome emphasizes PU as a facilitator of the combination of TAM and TPB in demonstrating the way that perceptions of functionality may be converted into long-lasting intentions in smart cities (Venkatesh and Davis, 2000).

PU is an important determinant of NZE, and perceived usefulness is crucial in users’ understanding and use of net-zero emissions. This is consistent with previous research (Bedimo-Rung et al., 2005), as perceived usefulness is extremely salient when it comes to the sharing economy, specifically in the use and understanding of shared bicycles (Xue et al., 2022). This again indicates the salience of environmental factors in influencing behavior intentions to utilize environmentally friendly transportation means like shared E-bikes. Therefore, users’ attitude and experience with shared bikes to a great extent can predict users’ evaluation of the usefulness of shared electric bikes. The correlation indicates not only the importance of user cognition in promoting friendly environmentally travel modes but also suggests the feasibility of using PU to promote low-carbon travel and aiding city sustainability efforts.

Empirical studies confirm that NZE has a significant and positive impact on PBC. The studies determined that environmental consciousness positively enhances the use of shared E-bicycles (Chen, 2022). Environmental consciousness and knowledge of net-zero emissions in the public can trigger the adoption of sustainable development strategies (Flores and Moore, 2024). NZE sensitizes individuals to be responsible for protecting the environment, hence influencing PBC and enacting pro-environmental actions, such as the use of E-bikes. E-bikes efficiently reduce greenhouse gases by replacing cars for short- to medium-distance journeys, which aligns with the vision of smart cities of low-carbon development. Their zero-emission ability not only aligns with net-zero goals but also provides cleaner air, which has a positive impact on urban ecosystems and the well-being of citizens.

To realize the full effect of this positive impact, policymakers and city planners must place utmost priority on awareness-raising initiatives and incentive systems that not only make citizens aware of net-zero goals but also translate such awareness into actual control and implementation of behavior (Steg and Vlek, 2009). Focused interventions, like the integration of E-bike infrastructure into public mobility networks and financial or social incentives for low-emission commuting, have the potential to bridge the gap between environmental awareness and effective behavioral change. However, translating initial awareness into long-term behavior is challenging, as it entails the positive reinforcement of initial behavior and the identification of follow-up behavior as naturally occurring positive reinforces (Zhao et al., 2024).

The research findings of this study have close connections with the SDGs and long-term future of smart city development. Green transportation shared E-bikes have close connections with a series of SDGs, including SDG 3 (ensure healthy lives), SDG 7 (ensure access to affordable, reliable, sustainable, and modern energy), SDG 9 (build resilient infrastructure and promote sustainable industrialization), SDG 11 (make cities inclusive, safe, resilient, and sustainable), and SDG 13 (take urgent action to combat climate change; Sharifi et al., 2024; Nieuwenhuijsen, 2020). Shared E-bikes not only enhance the health of city residents but also reduce carbon emissions, enhance urban energy efficiency, and promote sustainable urban development.

In addition, the utilization of E-bikes encourages light physical exercise that contributes to better health and potentially a reduced need for healthcare, reflecting the smart city vision of improving quality of life and encouraging healthier living.

Nevertheless, there are several limitations to this research that should be mentioned. Firstly, the sample was composed mostly of young respondents from coastal areas, which may limit the generalizability of the findings to broader populations. Secondly, the study did not consider potential moderating or mediating variables, i.e., income, environmental literacy, or prior E-bike use experience, which could potentially explain further variation in behavior. Thirdly, the reliance on cross-sectional survey data confines the extent to which changes in behavior can be measured over time. Future research can overcome these constraints by employing more representative and heterogeneous samples, incorporating more variables into the model, and employing longitudinal or experimental designs to capture more dynamic behavioral change. These improvements would yield further insights into shared E-bike adoption in heterogeneous urban environments and inform the design of more effective sustainable mobility policies.

## 6 Conclusion

This study rigorously investigated the role of subjective norms (SN), perceived usefulness (PU), use attitudes (ATU), net-zero emissions (NZE), and perceived behavioral control (PBC) on the attitudes and behavioral intentions of shared electric bike riders. The findings indicate that peer attitudes and social community values in the social environment positively support users’ PU, confirming social influence as the dominant force of user cognition under the sharing economy context. PU as the main mediator significantly influences users’ ATU and environmental sustainability perception and demonstrates high awareness of the convenience and environmental sustainability of shared E-bikes.

Besides, NZE not only supports users’ green consciousness but also increases usage intention by empowering PBC, which shows the instrumental role of green awareness in green mobility. PBC reflects users’ efficacy and control of behavior, and it is a critical determinant for general acceptance of shared E-bikes.

This research bridges a theoretical gap in existing smart city literature regarding the concurrent processes of social influence and environmental awareness. In using TAM and TPB together, it establishes PU as a link between SN and environmental awareness and users’ ATU and usage intent, thus extending the theoretical domains of these fundamental models. Significantly, not only does this extension of the model contribute to theory, but it also sets the platform for future research in further building the understanding of green travel option choice in the context of the sharing economy.

Covering China’s four major first-tier cities and several major and mid-sized inland cities, the sample is diversified but skewed towards youth. Future follow-up studies must enlarge the sample to other age and social strata so that the generality and robustness of the model can be tested with further evidence. Physical environment characteristics of shared E-bikes, such as infrastructure completeness, parking convenience, and transportation network connectivity, can exert significant impacts on users’ intention to adopt them again and should be incorporated into future theories.

Besides, as shared mobility policy and technology continue to evolve, longitudinal study designs can be employed to track evolving user behavior and attitudes in the long term and identify the processes and drivers of long-term take-up. This will provide a scientific foundation and actionable insight for green mobility policy-making and urban transport planning in smart cities.

Overall, this research not only enhances the understanding of user behavior towards shared electricity bicycles, but also offers theoretical and practical references for the development of sustainable urban transportation. It is hoped that future research will further enrich and refine the theoretical system for realizing the long-term goal of low-carbon smart cities.

## Data Availability

The raw data supporting the conclusions of this article will be made available by the authors, without undue reservation.

## References

[ref1] Ajzen I. (1991). The theory of planned behavior. Organ. Behav. Hum. Decis. Process. 50, 179–211.

[ref2] Ajzen I. Fishbein M. (1975). A bayesian analysis of attribution processes. Psychol. Bull. 82, 261–277.

[ref3] Ariff M. S. M. Yeow S. M. Zakuan N. Jusoh A. Bahari A. Z. (2012). The effects of computer self-efficacy and technology acceptance model on behavioral intention in internet banking systems. Procedia Soc. Behav. Sci. 57, 448–452. doi: 10.1016/j.sbspro.2012.09.1210

[ref4] Arkorful V. E. (2022). Unravelling electricity theft whistleblowing antecedents using the theory of planned behavior and norm activation model. Energy Policy 160:112680. doi: 10.1016/j.enpol.2021.112680

[ref5] Bagdatli M. E. C. Ipek F. (2022). Transport mode preferences of university students in post-COVID-19 pandemic. Transp. Policy 118, 20–32. doi: 10.1016/j.tranpol.2022.01.017, PMID: 35125682 PMC8799352

[ref6] Bai L. Sze N. N. Liu P. Haggart A. G. (2020). Effect of environmental awareness on electric bicycle users’ mode choices. Transp. Res. Part D Transp. Environ. 82:102320. doi: 10.1016/j.trd.2020.102320

[ref7] Bardi A. Mantecchini L. Grasso D. Paganelli F. Malandri C. (2019). Flexible mobile hub for E-bike sharing and cruise tourism: a case study. Sustainability 11:5462. doi: 10.3390/su11195462

[ref8] Bedimo-Rung A. L. Mowen A. J. Cohen D. A. (2005). The significance of parks to physical activity and public health: a conceptual model. Am. J. Prev. Med. 28, 159–168. doi: 10.1016/j.amepre.2004.10.024 15694524

[ref9] Buabeng-Andoh C. (2018). Predicting students’ intention to adopt mobile learning: a combination of theory of reasoned action and technology acceptance model. J. Res. Innov. Teach. Learn. 11, 178–191. doi: 10.1108/JRIT-03-2017-0004

[ref10] Campbell A. A. Cherry C. R. Ryerson M. S. Yang X. (2016). Factors influencing the choice of shared bicycles and shared electric bikes in Beijing. Transp. Res. Part C, Emerg. Technol. 67, 399–414. doi: 10.1016/j.trc.2016.03.004

[ref11] Chen X. (2022). Predicting college students’ bike-sharing intentions based on the theory of planned behavior. Front. Psychol. 13:836983. doi: 10.3389/fpsyg.2022.836983, PMID: 35310235 PMC8928168

[ref12] Cherry C. R. Weinert J. X. Xinmiao Y. (2009). Comparative environmental impacts of electric bikes in China. Transp. Res. Part D Transp. Environ. 14, 281–290. doi: 10.1016/j.trd.2008.11.003

[ref13] Choi G. Chung H. (2013). Applying the technology acceptance model to social networking sites (SNS): impact of subjective norm and social capital on the acceptance of SNS. Int. J. Hum.-Comput. Interact. 29, 619–628. doi: 10.1080/10447318.2012.756333

[ref14] Cruz S. S. Paulino S. R. (2022). Experiences of innovation in public services for sustainable urban mobility. J. Urban Manag. 11, 108–122. doi: 10.1016/j.jum.2021.10.003

[ref15] Davis F. D. Bagozzi R. P. Warshaw P. R. (1989). User acceptance of computer technology: a comparison of two theoretical models. Manag. Sci. 35, 982–1003.

[ref16] Daziano R. A. Bolduc D. (2013). Incorporating pro-environmental preferences towards green automobile technologies through a Bayesian hybrid choice model. Transp. A Transp. Sci. 9, 74–106. doi: 10.1080/18128602.2010.524173

[ref17] Duong H. N. Chu M. C. Huynh N. (2024). “An application of the theory of planned behavior to study red-light running behavior of adolescent riders in Ho Chi Minh City, Vietnam” in Proceedings of the Third International Conference on Sustainable Civil Engineering and Architecture. eds. Reddy J. N. Wang C. M. Luong V. H. Le A. T. (Singapore: Springer Nature), 1739–1747.

[ref18] Flores R. M. Moore T. A. (2024). Coal and coalbed gas: Future directions and opportunities. Amsterdam and Cambridge: Elsevier.

[ref19] Fornell C. Larcker D. F. (1981). Evaluating structural equation models with unobservable variables and measurement error. J. Mark. Res. 18, 39–50.

[ref20] Galatoulas N.-F. Genikomsakis K. N. Ioakimidis C. S. (2020). Spatio-temporal trends of e-bike sharing system deployment: a review in Europe, North America and Asia. Sustainability 12:4611. doi: 10.3390/su12114611

[ref21] Hair J. Anderson R. Babin B. Black W. (2013). Multivariate Data Analysis. Harlow, Essex, England: Pearson International.

[ref22] Horstmannshoff T. Redmond M. (2024). Identifying alternative stops for first and last-mile urban travel planning. Public Transp. 16, 359–379. doi: 10.1007/s12469-024-00355-w

[ref23] Jones T. Harms L. Heinen E. (2016). Motives, perceptions and experiences of electric bicycle owners and implications for health, wellbeing and mobility. J. Transp. Geogr. 53, 41–49. doi: 10.1016/j.jtrangeo.2016.04.006

[ref24] Junaid M. Ferretti M. (2024). “Planning and designing a sustainable mobility system in rural areas” in Urban climate change adaptation. eds. He B. Jupesta J. Cirella G. T. Pignatta G. (Cham, Switzerland: Springer Nature Switzerland), 205–228.

[ref25] Kazemzadeh K. Ronchi E. (2022). From bike to electric bike level-of-service. Transp. Rev. 42, 6–31. doi: 10.1080/01441647.2021.1900450

[ref26] Kippax S. Crawford J. (1993). “Flaws in the theory of reasoned action” in The theory of reasoned action. eds. Gallois C. McCamish M. Terry D. J. (London: Garland Science). 10.1111/j.2044-8309.1993.tb00997.x 8220941

[ref27] Kline R. B. (2023). Principles and practice of structural equation modeling. New York, NY: Guilford Publications.

[ref28] Küfeoğlu S. (2024). “Transportation sector emissions” in Net zero decarbonizing the global economies (Cham: Springer), 493–554.

[ref32] Li L. Zhu B. Jiang M. Cai X. Lau A. K. W. Shin G.-C. (2020). The role of service quality and perceived behavioral control in shared electric bicycle in China: does residual effects of past behavior matters? Environ. Sci. Pollut. Res. 27, 24518–24530. doi: 10.1007/s11356-020-08690-8, PMID: 32306269

[ref29] Li R. Krishna Sinniah G. Li X. (2022). The factors influencing resident’s intentions on e-bike sharing usage in China. Sustainability 14:5013. doi: 10.3390/su14095013

[ref30] Li W. Yang Y. Cheng L. Meng X. Zhang F. Ji Y. (2023). Understanding adoption intent and behavioral response to shared electric bicycles: a survey in Ningbo, China. Transp. Res. Rec. 2677, 1311–1326. doi: 10.1177/03611981221103874

[ref31] Li X. Zhang Y. Yang Z. Zhu Y. Li C. Li W. (2023). Modeling choice behaviors for ridesplitting under a carbon credit scheme. Sustainability 15:12241. doi: 10.3390/su151612241

[ref33] MacArthur J. Kobel N. Dill J. Mumuni Z. (2017). Evaluation of an electric bike pilot project at three employment campuses. Portland, Oregon: TREC Final Reports. doi: 10.15760/trec.158

[ref34] Makido Y. Dhakal S. Yamagata Y. (2012). Relationship between urban form and CO2 emissions: evidence from fifty Japanese cities. Urban Clim. 2, 55–67. doi: 10.1016/j.uclim.2012.10.006

[ref35] Moan I. S. Rise J. (2006). Predicting smoking reduction among adolescents using an extended version of the theory of planned behaviour. Psychol. Health 21, 717–738. doi: 10.1080/14768320600603448

[ref36] Nieuwenhuijsen M. J. (2020). Urban and transport planning pathways to carbon neutral, liveable and healthy cities; a review of the current evidence. Environ. Int. 140:105661. doi: 10.1016/j.envint.2020.105661, PMID: 32307209

[ref37] Ogunbode C. A. Demski C. Capstick S. B. Sposato R. G. (2019). Attribution matters: revisiting the link between extreme weather experience and climate change mitigation responses. Glob. Environ. Chang. 54, 31–39. doi: 10.1016/j.gloenvcha.2018.11.005

[ref38] Pan L. Xia Y. Xing L. Song Z. Xu Y. (2022). Exploring use acceptance of electric bicycle-sharing systems: an empirical study based on PLS-SEM analysis. Sensors 22:7057. doi: 10.3390/s22187057, PMID: 36146406 PMC9503645

[ref39] Pasca M. G. Guglielmetti Mugion R. Di Pietro L. Renzi M. F. (2024). Unveiling the role of gamification in shared mobility services. Environ. Dev. Sustain. 27, 13371–13410. doi: 10.1007/s10668-024-04465-0

[ref40] Pierce J. T. Nash A. B. Clouter C. A. (2013). The in-use annual energy and carbon saving by switching from a car to an electric bicycle in an urban UK general medical practice: the implication for NHS commuters. Environ. Dev. Sustain. 15, 1645–1651. doi: 10.1007/s10668-013-9454-0

[ref41] Rehman Z. U. Seman N. A. A. Harun A. (2024). Exploring intention to purchase green products using the theory of reasoned action: testing the moderating effect of price sensitivity. Process Integr. Optim. Sustain. 8, 1649–1662. doi: 10.1007/s41660-024-00451-1

[ref42] Rezvani Z. Jansson J. Bodin J. (2015). Advances in consumer electric vehicle adoption research: a review and research agenda. Transp. Res. Part D Transp. Environ. 34, 122–136. doi: 10.1016/j.trd.2014.10.010

[ref43] Ricci M. (2015). Bike sharing: a review of evidence on impacts and processes of implementation and operation. Res. Transp. Bus. Manag. 15, 28–38. doi: 10.1016/j.rtbm.2015.03.003

[ref44] Samet B. Couffin F. Zolghadri M. Barkallah M. Haddar M. (2018). Performance analysis and improvement of the bike sharing system using closed queuing networks with blocking mechanism. Sustainability 10:4663. doi: 10.3390/su10124663, PMID: 40771761

[ref45] Samuel M. Doctor G. Christian P. Baradi M. (2020). Drivers and barriers to e-government adoption in Indian cities. J. Urban Manag. 9, 408–417. doi: 10.1016/j.jum.2020.05.002

[ref46] Shakya L. K. Devkota N. Dhakal K. Poudyal R. Mahato S. Paudel U. R.. (2024). Consumer’s behavioural intention towards adoption of e-bike in Kathmandu valley: structural equation modelling analysis. Environ. Dev. Sustain. 27, 16237–16265. doi: 10.1007/s10668-024-04595-5

[ref47] Sharifi A. Allam Z. Bibri S. E. Khavarian-Garmsir A. R. (2024). Smart cities and sustainable development goals (SDGs): a systematic literature review of co-benefits and trade-offs. Cities 146:104659. doi: 10.1016/j.cities.2023.104659

[ref48] Shi J. Jiang Z. (2023). Willingness to pay a premium price for green products: does a reference group matter? Environ. Dev. Sustain. 25, 8699–8727. doi: 10.1007/s10668-022-02419-y

[ref49] Song H. Yin G. Wan X. Guo M. Xie Z. Gu J. (2022a). Increasing bike-sharing users’ willingness to pay—a study of China based on perceived value theory and structural equation model. Front. Psychol. 12. doi: 10.3389/fpsyg.2021.747462, PMID: 35115981 PMC8805152

[ref50] Song H. Zeng W. Zeng T. (2022b). Modeling community residents’ exercise adherence and life satisfaction: an application of the influence of the reference group. Int. J. Environ. Res. Public Health 19:13174. doi: 10.3390/ijerph192013174, PMID: 36293754 PMC9603160

[ref51] Standing C. Standing S. Biermann S. (2019). The implications of the sharing economy for transport. Transp. Rev. 39, 226–242. doi: 10.1080/01441647.2018.1450307

[ref52] Steg L. Vlek C. (2009). Encouraging pro-environmental behaviour: an integrative review and research agenda. J. Environ. Psychol. 29, 309–317. doi: 10.1016/j.jenvp.2008.10.004

[ref53] Su J. Luo S. Ji K. Tian B. (2024). Perceived value, service quality and behavioral intentions towards bike-sharing services: Using an extended technology acceptance model. Service quality and behavioral intentions towards bike-sharing services: Using an extended technology acceptance model. Res. Transp. Bus. Manag. 57:101236. doi: 10.1016/j.rtbm.2024.101236

[ref54] Sweeney S. Ordóñez-Hurtado R. Pilla F. Russo G. Timoney D. Shorten R. (2018). A context-aware e-bike system to reduce pollution inhalation while cycling. IEEE Trans. Intell. Transp. Syst. 20, 704–715. doi: 10.1109/TITS.2018.2825436

[ref56] Tee M. Al Mamun A. Salameh A. A. (2023). Modelling the mass adoption potentials of eBikes among Malaysian youth. Environ. Sci. Pollut. Res. 30, 95475–95492. doi: 10.1007/s11356-023-29129-w, PMID: 37548792

[ref57] Teo T. Lee C. B. Chai C. S. (2008). Understanding pre-service teachers’ computer attitudes: applying and extending the technology acceptance model. J. Comput. Assist. Learn. 24, 128–143. doi: 10.1111/j.1365-2729.2007.00247.x

[ref58] Teo T. Van Schaik P. (2012). Understanding the intention to use technology by preservice teachers: an empirical test of competing theoretical models. Int. J. Hum.-Comput. Interact. 28, 178–188. doi: 10.1080/10447318.2011.581892

[ref59] Venkatesh V. Davis F. (2000). A theoretical extension of the technology acceptance model: Four longitudinal field studies. Manage. Sci. 46, 186–204. doi: 10.1287/mnsc.46.2.186.11926

[ref60] Wang P. Priyadarshi R. (2024). Policy framework for realizing net-zero emission in smart cities. Arch. Comput. Methods Eng. 32, 63–82. doi: 10.1007/s11831-024-10131-5

[ref9001] Wilson M. Whitmarsh L. (2025). Behavior change interventions to promote adoption of e-bike shared mobility in a rural area: Evidence from a mixed-method field trial. Front. Psychol. 16:1569176. doi: 10.3389/fpsyg.2025.1569176 40463296 PMC12131865

[ref9002] World Medical Association. (2013). World medical association declaration of Helsinki: Ethical principles for medical research involving human subjects. JAMA. 310, 2191–2194. doi: 10.1001/jama.2013.281053 24141714

[ref9003] Xu C. Ma Q. Lu Y. Liang Q. Gao W. (2023). Improving cycling environment in a Green Park based on the post-occupancy evaluation method. J. Asian Archit. Build. Eng. 22, 513–529. doi: 10.1080/13467581.2022.2046596

[ref61] Xue X. Wang Z. Liu X. Zhou Z. Song R. (2022). A choice behavior model of bike-sharing based on user perception, psychological expectations, and loyalty. J. Adv. Transp. 2022:6695977. doi: 10.1155/2022/6695977

[ref62] Yang W. Li T. Cao X. (2015). Examining the impacts of socio-economic factors, urban form and transportation development on CO2 emissions from transportation in China: a panel data analysis of China’s provinces. Habitat Int. 49, 212–220. doi: 10.1016/j.habitatint.2015.05.030

[ref64] Zhang X. Manogaran G. Muthu B. (2021a). IoT enabled integrated system for green energy into smart cities. Sustain Energy Technol Assess 46:101208. doi: 10.1016/j.seta.2021.101208

[ref65] Zhang X. Wang J. Long X. Li W. (2021b). Understanding the intention to use bike-sharing system: a case study in Xi’an, China. PLoS One 16:e0258790. doi: 10.1371/journal.pone.0258790, PMID: 34855753 PMC8638983

[ref63] Zhang Y. Li C. Ding C. Zhao C. Huang J. (2016). The built environment and the frequency of cycling trips by urban elderly: insights from Zhongshan, China. J. Asian Archit. Build. Eng. 15, 511–518. doi: 10.3130/jaabe.15.511

[ref66] Zhao J. Radke J. Chen F. S. Sachdeva S. Gershman S. J. Luo Y. (2024). How do we reinforce climate action? Sustain. Sci. 19, 1503–1517. doi: 10.1007/s11625-024-01486-6

[ref67] Zhu M. Hu X. Lin Z. Li J. Wang S. Wang C. (2020). Intention to adopt bicycle-sharing in China: introducing environmental concern into the theory of planned behavior model. Environ. Sci. Pollut. Res. 27, 41740–41750. doi: 10.1007/s11356-020-10135-1, PMID: 32691322

